# Whole-Exome Sequencing to Identify a Novel LMNA Gene Mutation Associated with Inherited Cardiac Conduction Disease

**DOI:** 10.1371/journal.pone.0083322

**Published:** 2013-12-12

**Authors:** Chun-Chi Lai, Yung-Hsin Yeh, Wen-Ping Hsieh, Chi-Tai Kuo, Wen-Ching Wang, Chia-Han Chu, Chiu-Lien Hung, Chia-Yang Cheng, Hsin-Yi Tsai, Jia-Lin Lee, Chuan-Yi Tang, Lung-An Hsu

**Affiliations:** 1 Department of Computer Science, National Tsing Hua University, Hsinchu, Taiwan; 2 First Cardiovascular Division, Chang Gung Memorial Hospital, Chang Gung University College of Medicine, Tao-Yuan, Taiwan; 3 Institute of Statistics, National Tsing Hua University, Hsinchu, Taiwan; 4 Institute of Molecular and Cellular Biology and Department of Life Sciences, National Tsing-Hua University, Hsinchu, Taiwan; 5 Biomedical Science and Engineering Center, National Tsing Hua University, Hsinchu, Taiwan; Yale School of Public Health, United States of America

## Abstract

**Background:**

Inherited cardiac conduction diseases (CCD) are rare but are caused by mutations in a myriad of genes. Recently, whole-exome sequencing has successfully led to the identification of causal mutations for rare monogenic Mendelian diseases.

**Objective:**

To investigate the genetic background of a family affected by inherited CCD.

**Methods and Results:**

We used whole-exome sequencing to study a Chinese family with multiple family members affected by CCD. Using the pedigree information, we proposed a heterozygous missense mutation (c.G695T, Gly232Val) in the lamin A/C (*LMNA*) gene as a candidate mutation for susceptibility to CCD in this family. The mutation is novel and is expected to affect the conformation of the coiled-coil rod domain of LMNA according to a structural model prediction. Its pathogenicity in lamina instability was further verified by expressing the mutation in a cellular model.

**Conclusions:**

Our results suggest that whole-exome sequencing is a feasible approach to identifying the candidate genes underlying inherited conduction diseases.

## Introduction

The functional components of the cardiac conduction system can be broadly divided into the impulse-generating nodes and the impulse-propagating His-Purkinje system. In cardiac conduction disease (CCD), the integrity of the conduction system is impaired such that impulse generation, impulse propagation, or both will be slowed or even blocked and life-threatening rhythm disturbances may ensue. CCD may result from acquired injury such as ischemia or drug toxicity, be associated with heart diseases such as congenital defects or cardiomyopathy, be associated with neuromuscular disease, or rarely be an isolated finding [1,2]. The familial clustering of idiopathic CCD has led to the discovery of a large number of mutations identified in genes encoding ion channels (Voltage-Gated Sodium Channel Subunit Alpha (*SCN5A*), Hyperpolarization Activated Cyclic Nucleotide-Gated Potassium Channel 4 (*HCN4*), Potassium Inwardly-Rectifying Channel Subfamily J Member 2 (*KCNJ2*)), cardiac transcription factors (Homeobox protein Nkx-2.5 (*NKX2-5*), T-box transcription factor (*TBX5*)), gap junctions (Connexin 40 (*Cx40*)), energy metabolism regulators (5'-AMP-activated protein kinase subunit gamma-2 (*PRKAG2*)) and structural proteins (lamin A/C (*LMNA*)) that cause progressive conduction system disease in the absence of structural heart disease [1,2]. Inherited forms of CCD are rare, but each new mutation provides helpful insights into the molecular mechanisms controlling cardiac conduction system development and function [3]. 

Clinical genetic testing can help identify patients at risk for CCD before its manifestation. However, genetic testing techniques, knowledge about the candidate genes and the size of the pedigrees may compromise the ability to uncover relevant genetic defects. The analysis is often restricted to previously identified candidate genes and may fail to uncover previously unidentified causal genes for familial CCD. Linkage analysis may be compromised by small pedigree sizes. Therefore, next-generation whole-genome or exome sequencing may help overcome these limitations.

Many of the recent studies analyzed variant data from exome sequencing to identify causal genes for Mendelian diseases [4-8]. Exome sequencing is a powerful tool for characterizing DNA sequences surrounding target regions at a much lower cost compared to the whole-genome sequencing technique [9]. In some cases, researchers have analyzed exome sequencing data from only one patient [4,5], whereas most studies have used exome sequencing data from several members of an afflicted family [6-8]. Some studies have summarized the computational workflow from those reports [10,11]. In this study, we investigated a Chinese family with inherited CCD that was characterized by sinus arrest, atrial fibrillation and atrioventricular conduction disturbance. Using emerging whole-exome sequencing techniques, we identified a novel point mutation located in *LMNA*, which encodes the inner nuclear membrane protein lamin A/C. Structural modeling predicts that this mutation may affect *LMNA* function. Cellular modeling further verifies its pathogenicity in lamina stability. 

## Methods

### Ethics Statement

The protocols were approved by the Human Research Ethics Committee at Chang Gung Memorial Hospital (Chang Gung Medical Foundation Institutional Review Board 99-2468B) and were conducted in concordance with the Declaration of Helsinki Principles. Written informed consent was obtained from each subject.

### Study Population

**Figure 1:**
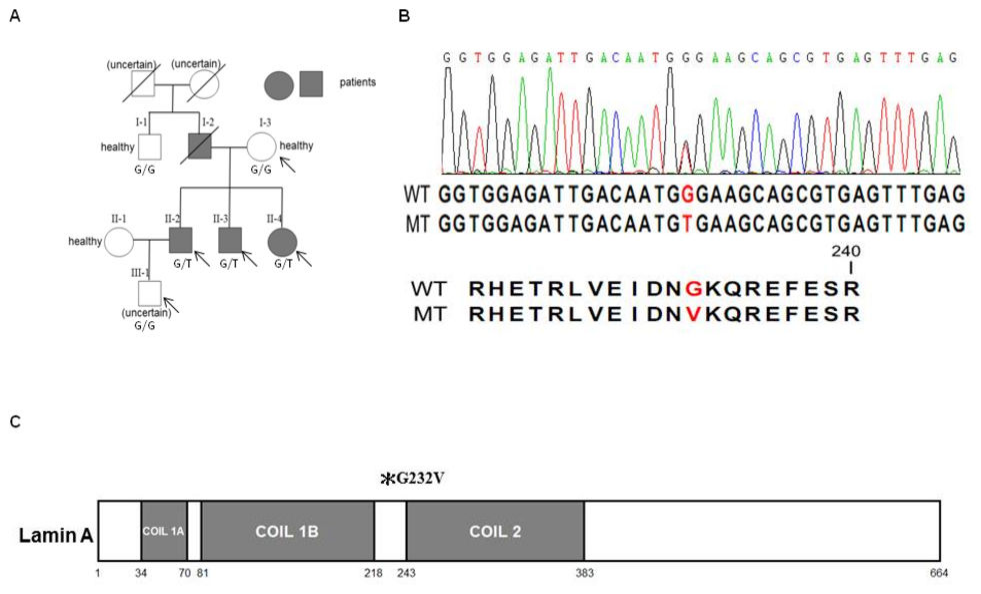
Novel *LMNA* mutation (G232V) segregates with inherited cardiac conduction disease. (**A**) Pedigree of the studied family. The pedigree shows the affection statuses, individual identifiers, and genotypes at *LMNA* c.G695T. The samples (marked by arrows) I-3, II-2, II-3, II-4 and III-1 were exome sequenced. The I-1, I-3 and II-1 samples were from healthy individuals, and the phenotype of sample III-1 was uncertain. (**B**) Sequencing result showing the heterozygous *LMNA* c.G695T (G232V) mutation. WT, wild type allele; MT, mutant allele. (**C**) Schematic of Lamin A protein. *G232V mutation in the 1B−2 linker region.

Genomic DNA was prepared from the venous blood of a variety of affected and unaffected individuals following standard procedures. The first set of analyses was performed on a Chinese family with inherited CCD (Family TS; pedigree in Figure 1A). CCD was characterized by early onset, symptomatic arrhythmia, including a long pause after atrial fibrillation and a combination of sino-atrial and atrioventricular nodal conduction blocks, which led to pacemaker implantation between the ages of 44 and 64. The study family consisted of one affected father, three affected siblings, one unaffected mother, one unaffected uncle and a grandson with an uncertain phenotype. The validation study included a cohort of 252 additional unrelated controls recruited from a population receiving routine health examinations and a population receiving regular hypertension treatment in an outpatient clinic. Among them, 137 subjects displayed sinus rhythm and 115 subjects displayed atrial fibrillation.

### Clinical Assessment

Clinical data, including medical history, physical examination, 12-lead electrocardiogram (ECG), 24-hour Holter recordings and echocardiographic data on the study family, were collected and evaluated (Table 1). All affected family members (the father and 3 siblings; I-2, II-2, II-3 and II-4) presented with a combination of at least two of the following phenotypes: sinus nodal dysfunction, paroxysmal atrial fibrillation, or atrioventricular conduction block. Three affected family members (the father and 2 brothers; I-2, II-2, and II-3) had permanently implanted pacemakers. The diagnosis of sinus node dysfunction was also confirmed by an electrophysiology study in two affected members (II-3 and II-4). Sinus node dysfunction was defined as sinus bradycardia, sino-atrial exit block and/or sinus pause or arrest. The echocardiographic findings from the 4 affected family members showed normal left and right ventricular sizes and contractile function (LV ejection fraction >50%). None of the findings indicated significant valvular disease. The affected father died from a stroke at the age of 78 and was screened for a mutation in the *SCN5A* gene before this study. He did not carry any mutations in the entire protein-coding region or the intron/exon boundaries of the *SCN5A* gene, and his DNA was not available for further exome sequencing. The grandson, whose phenotype was not established, was enrolled at the age of 31 and did not show abnormal findings in a 12-lead ECG, 24-hour Holter recordings, or echocardiography examination. The unaffected uncle was enrolled at the age of 85 and did not show abnormal findings in a 12-lead ECG and echocardiography examination.

**Table 1:** Demographic and phenotypic information on the study family.

	Sex	Conduction defect	Paf	Age at echo	LV EF, %	LA, mm	LV, mm	Age at death	Age at PPM
I-1	M	No	No	85	66	36	56		
I-2	M	SND, 2^nd^-degree AVB	yes	64	76	35	41	78	64
I-3	F	No	No	73	58	34	34		
II-2	M	SND, 3^rd^-degree AVB	yes	48	70	34	49		44
II-3	M	SND, 1^st^- & 2^nd^-degree AVB	yes	45	69	36	48		48
II-4	F	SND	yes	44	74	29	40		
III-1	M	No	No	31	77	36	48		

Paf, paroxysmal atrial fibrillation; SND, sinus node dysfunction; AVB, atrioventricular block; PPM, permanent pacemaker implantation

### Exome Sequencing

Three micrograms of DNA from each of the 5 family members (the 3 affected siblings, the healthy mother and the grandson) were subjected to exome enrichment with the TruSeq Exome Enrichment Kit, according to the protocol provided by the manufacturer. The Illumina TruSeq Enrichment kit was designed to target 201,121 exons within the 20,794 human gene regions. The kit allowed us to target 62.1 million bases of the human genome and enabled the capture of 97.2% of the exons defined by the consensus coding sequence (CCDS). Enriched DNA libraries were then sequenced on an Illumina Genome Analyzer IIx at the Biomedical Science and Engineering Center, National Tsing Hua University, Hsinchu, Taiwan. The original data files have been deposited in the NIH Short Read Archive (http://www.ncbi.nlm.nih.gov/sra) under the accession number SRP030761.

### Sequence Alignment and Variant Calling

To maximize the information retrieved and improve confidence in the results, we used the Burrows-Wheeler Alignment tool (BWA) [12] as a second alignment tool in addition to the Consensus Assessment of Sequence and Variation (CASAVA) default tool for the sequencing system. The poor quality short reads were filtered out and aligned to the human reference genome (NCBI Build 37/hg19). The Sequence Alignment and Mapping (SAM) files were converted to Binary Alignment and Mapping files (BAM) using SAMtools [13]. Picard (http://picard.sourceforge.net/) was used to mark and remove the polymerase chain reaction (PCR) duplicates detected from the BAM files. The Genome Analysis Toolkit (GATK) [14] was then used for base quality recalibration and local realignment around the potential insertion/deletion (Indel) sites. The UnifiedGenotyper in the GATK was used in the final step for variant calling using the Bayesian model. The loci with more than 3 alleles were filtered, and the bi-allelic variants matching any of the three inheritance models in the subsequent step were reserved to pass through the workflow. 

### Matching Genotypes with Pedigree Analysis

**Figure 2:**
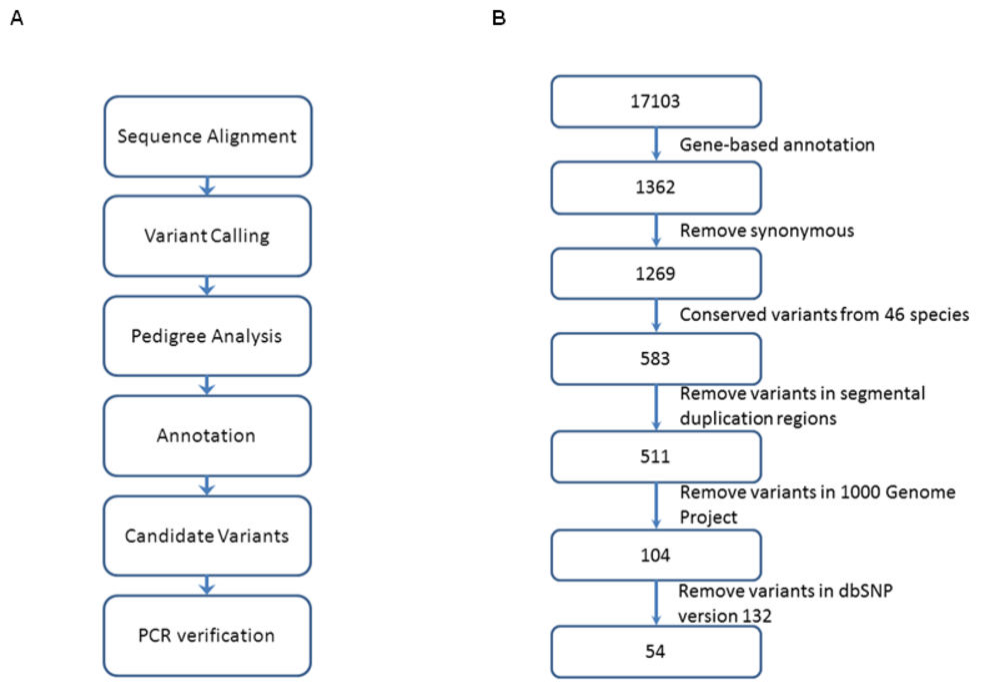
Workflow and annotation pipeline for the identification of candidate variants. (**A**) Workflow for finding candidate variants. (**B**) The number of SNV and Indel variants from GATK-called data sorted following the autosomal dominant model through the ANNOVAR pipeline. There were 52 SNV and 2 Indel variants remaining, and each variant was located in different genes.

Figure 2A illustrates the workflow for the identification of candidate variants. The first filtering step was performed to infer the genotypes of each family member from the pedigree according to the possible inheritance models (Figure S1). We filtered out the variants that did not match any reasonable inheritance pattern. One of the affected members (sample II-4) was female, so the Y chromosome was excluded. The mitochondrial chromosome was excluded because mitochondria are transmitted almost exclusively through the mother. X-linked dominant inheritance was also excluded by the male-to-male transmission. Although autosomal dominant inheritance is the most likely transmission model, we also analyzed variants matching the criteria for the autosomal recessive and X-linked recessive inheritance models.

### Annotation Analysis

Variants that passed the pedigree analysis were annotated using ANNOVAR [15] for function (exonic or splicing); gene; exonic function (synonymous, nonsynonymous, stop gain, nonframeshift or frameshift indel); amino acid change; conservation; allele frequency in ESP 5400 Exome Project; dbSNP (version 132) reference number; allele frequency in 1000 Genomes Project (2012 February version); dbNSFP [16] functional prediction scores that integrated and normalized scores for PhyloP, SIFT, Polyphen2, LRT and GERP++; and chromosome position. ANNOVAR processed the combined variants with the three transmission models. The first step in the pipeline was to extract and annotate single nucleotide variants (SNVs) in the exonic regions and then to exclude the synonymous variants (Figure 2B). ANNOVAR then selected the variants located in the genomic regions that are highly conserved among 46 vertebrate model organisms. The SNVs and Indels that appeared in the segmental duplication regions, dbSNP and the 1000 Genomes Project were subsequently filtered out. We used a maximal minor allele frequency (MAF) <0.05 filter for the 1000 Genome project because our target was a rare variant. The effects of the remaining nonsynonymous SNVs on protein function were then predicted using dbNSFP [16] and SIFT 2013 (updated version 5.0) [17].

### Mutation Validation

All of the candidate variants were reconfirmed by Sanger sequencing with the new PCR product for all 3 affected siblings, the healthy uncle, mother and grandson. Oligonucleotide primers were generated to amplify the fragments of genomic DNA containing identified variants (Table S1). The novel variants that were validated in the affected members were then examined in at least 100 unrelated healthy persons with normal phenotypes to exclude the possibility of polymorphisms. The genotyping was performed by PCR and restriction enzyme digestion, as described in Table S1.

### LMNA wild-type/mutation Structural Modeling

Homology detection and structural prediction by HMM-HMM comparison (HHpred) [18] was employed to build the structural models of wild-type (WT) and Gly232Val lamin A/C (*LMNA*) based on the crystal structures of intermediate filaments (tropomyosin from *Sus scrofa*, PDB ID: 1C1G [19]; human lamin coil 2B, PDB ID: 1X8Y [20]; human vimentin coil 2B fragment, PDB ID: 1GK4 [21] and human vimentin coil 2 fragment, PDB ID: 3TRT [22]). HHblits [23], a HMM-HMM-based lightning-fast iterative sequence search tool in the HHpred suite, was used to generate a multiple sequence alignment (MSA) profile between the templates and WT/mutant with a maximum of three MSA generation iterations. The structural models of WT LMNA and the G232V mutant (residues 81−383) were built from the generated MSA profile using Modeller [24]. 

### Expression Analysis

To characterize the consequences of the LMNA mutation at the cellular level, transient cell transfections were performed in HL-1 atrial cardiomyocytes with WT or mutated lamin A (NM_170707.3) and lamin C (NM_005572.3) mRNA expressed as fusions to the C-terminus of green fluorescent protein ( pCMV6-AC-GFP, RG204970 and RG201809 for Lamin A and Lamin C repectively, OriGene Technologies). Mutations were introduced via site-directed mutagenesis (Q5^®^Site-directed mutagenesis kit, New England Biolabs) with the forward primer 5′-GTGGAGATTGACAATG**T**GAAGCAGCGTGAGTTTG-3′ and the reverse primer 5′-CAAACTCACGCTGCTTC**A**CATTGTCAATCTCCAC-3′. All of the inserts were systematically verified by sequencing. HL-1 myocytes, a cell line derived from atria of adult mice, were cultured in Claycomb medium containing 10% fetal bovine serum, 1% penicillin/streptomycin, 2 mmol/L L-glutamine and 0.1 mmol/L norepinephrine and incubated at 37°C with 5% CO_2_. The transfection was performed by incubating 2 μg of fusion protein construct using LipofectAMINE 2000 (Invitrogen) according to the manufacturer’s instructions. Cells were grown for 48 hours. HL-1 images were captured using a Leica confocal laser-scanning microscope (Leica TCS SP2, Wetzlar, Germany).

## Results

### Exome Sequencing, Sequence Alignment and Variant Calling

We listed the number of short reads mapped to the reference genome (NCBI Build 37, hg19) for each sample in Table 2. Approximately 9.36 Gb of average sequence was generated per sample as 76-bp paired-end reads. Although only approximately 50% of the mapped reads were aligned to the target regions encompassing the exons, the average read depths in the target regions were much higher than those outside of the target regions. After removing the PCR-duplicated reads, 2.58 Gb of average sequence per sample mapped to the targeted exome sequences with a mean depth of 42.78-fold. On average, 87.02% of the exome was covered at least 10-fold. Overall, we identified approximately 381,264 SNVs and 34,937 Indels from 5 study samples (150,000 to 180,000 variants per subject) (Table 2). 

**Table 2:** Number of reads, variants and variant coverage by sample.

	Total reads number	Average read depth outside the target regions	Average read depth in the target regions	Mapping ratio outside the target regions	Mapping ratio in the target regions	Unmapped reads ratio
I-3	137213408	11.92	72.40	44.23%	48.96%	6.81%
II-2	141576846	12.37	78.61	41.65%	52.07%	6.28%
II-3	98710290	10.64	48.02	47.44%	45.22%	7.34%
II-4	130819190	12.26	68.47	45.41%	48.49%	6.10%
III-1	107741382	10.83	55.96	44.67%	48.29%	7.04%

### Variant filtration and prioritization according to inheritance models

Our first selection strategy was to find variants that matched possible inheritance models. When a genotyped variant from the 5 studied family members did not fit the assumed inheritance models, this gene was excluded from further investigation. As shown in Table 3, 15,875 SNVs and 1,228 Indels fit the autosomal dominant inheritance pattern. Only 2,070 SNVs and 195 Indels and 2 SNVs and 2 Indels fit the autosomal recessive and X-linked recessive inheritance patterns, respectively (Table 3).

**Table 3:** Number of combined SNVs and Indels remaining after each filtering step.

Step 0		Step 1			Step 2	
GATK calling		Inheritance model filtering			ANNOVAR filtering	
SNVs	381264	Autosomal Dominant	SNVs	15875	SNVs	52
Indels	34937		Indels	1228	Indels	2
		Autosomal Recessive	SNVs	2070	SNVs	0
			Indels	195	Indels	0
		X-linked Recessive	SNVs	2	SNVs	0
			Indels	2	Indels	0

### Annotation Analysis

All of the fitted variants were then subjected to ANNOVAR [15] for annotation. The ANNOVAR automatic pipeline filtered out most of the synonymous SNVs that were considered neutral variants and the nonsynonymous SNVs that were reported in the dbSNP135 and 1000 Genomes Project (MAF >0.05) databases. The variant numbers following the autosomal dominant inheritance model and remaining after each step of the ANNOVAR automatic pipeline are shown in Figure 2B. Only 52 SNVs and 2 Indels that matched the autosomal dominant model passed through the ANNOVAR pipeline (Table 3). In contrast, all of the variants sorted by the autosomal recessive or X-linked recessive models were excluded after ANNOVAR filtering (Table 3).

### Candidate Genes

The 2 remaining Indels were located in the chondroitin polymerizing factor 2 (*CHPF2*) and hephaestin-like 1 (*HEPHL1*) genes (Table S2). According to the Online Mendelian Inheritance in Man (OMIM) and PubMed databases, neither gene has been linked to heart disease. Of the 52 remaining SNVs (Table S3), we filtered out another 33 variants that had been reported in the 1000 Genomes Project (MAF <0.05) and the ESP5400 Exome Project and were considered known variants. Nineteen candidate variants remained and were entered into function prediction programs. Among them, only the *LMNA* mutation (c.G695T, p.Gly232Val, Figures 1B and 1C) was consistently predicted to be a deleterious mutation across both the dbNSFP [16] and SIFT 2013 programs (Table 4).

**Table 4:** Family-specific variants sorted by autosomal dominant inheritance and annotated by ANNOVAR.

SNV	PhyloP*	SIFT	PolyPhen	LRT**^*&*^**	MutationTaster**^*#*^**	GERP++	SIFT2013
SLC39A1	C	D	P	D	D	C	T
LMNA	C	D	D	D	D	C	D
SACM1L**^*†*^**	N/A	N/A	N/A	N/A	N/A	C	N/A
ADH4	N	D	D	N	D	C	D
MFSD8	N	T	P	N	D	N	T
DLK2	C	D	D	D	N	C	T
MDN1	C	T	B	D	N	C	T
ODZ4	N/A	N/A	N/A	N/A	N/A	C	B
ANGPTL5	C	T	D	D	D	C	T
L2HGDH	C	T	P	D	D	C	T
ZNF646	C	T	D	N/A	N/A	C	T
LONP2	C	T	D	D	D	C	T
MMP2	C	T	P	D	D	C	T
ZFHX3	C	N/A	P	D	D	C	T
MVD	C	D	B	D	D	C	T
KCNH6	C	T	D	D	D	C	T
USF2	C	T	B	N	N	C	T
MYH7B	C	N/A	N/A	N	N	C	T
ARHGAP40	N/A	N/A	N/A	N/A	N/A	C	T

*†* SACM1L intronic splicing site mutation;

*N, not conserved; C, conserved; D, deleterious; T, tolerated; P, possible damage; B, benign; **^*&*^**N, neutral; **^*#*^**N, polymorphism; N/A, not applicable;

Table S3 shows the detailed dbNSFP [16] functional prediction scores for each SNV.

### Validation of Candidate Genes

All of the 19 candidate variants, except the SAC1 Suppressor of Actin Mutations 1-Like (*SACM1L*) gene mutation, were missense mutations. We searched for potential links between the 19 candidate genes and cardiovascular diseases using the OMIM and PubMed databases. Six candidate genes including *LMNA*, Zinc Finger Homeobox 3 (*ZFHX3*), Matrix Metallopeptidase 2 (MMP2), Angiopoietin-Like 5 (*ANGPTL5*), Myosin Heavy Chain 7B (*MYH7B*) and Potassium voltage-gated channel subfamily H member 6 (*KCNH6*) have been previously linked to heart disease. Although the intronic splicing site mutation in the *SACM1L* gene has not been linked to heart disease, its effect on protein function could not be predicted by either dbNSFP or SIFT 2013. Therefore, we conducted direct sequencing validation for these 7 potential candidates in all 3 affected siblings, the mother, the uncle, the grandson and the 100 unrelated healthy controls. The *MYH7B* variant (Chr20:33588956 G/A) was present in 2 of the 100 controls and was thus considered a polymorphism. The *LMNA*, *ZFHX3*, *MMP2* and *ANGPTL5* SNVs were all novel and family-specific mutations (Table 5). The *LMNA* mutation was not present in the healthy mother, uncle and grandson. However, both of the *ZFHX3* and *MMP2* mutations were also present in the grandson, whose phenotype was not established and is most likely unaffected. Furthermore, the other three novel mutations, excluding *LMNA*, were predicted to be benign mutations. The *LMNA* gene remained the most likely candidate gene for CCD requiring further investigation. Additionally, both the *KCNH6* and *SACM1L* gene mutations were not validated by direct sequencing and were thus categorized as calling errors. 

**Table 5:** Candidate SNVs for a causal mutation in the family.

Gene Name	LMNA	MMP2	ZFHX3	ANGPTL5
SNV position	Chromosome 1, 156104651	Chromosome 16, 55519584	Chromosome 16, 72830334	Chromosome 11, 101773401
Variants	c.G695T, p.G232V	c.G577A, p.E193K	c.G3505A, p.V1169M	c.C491T, p.P164L
III-1 genotype	GG	GA	GA	CC

### LMNA wild-type/mutation Structural Modeling

**Figure 3:**
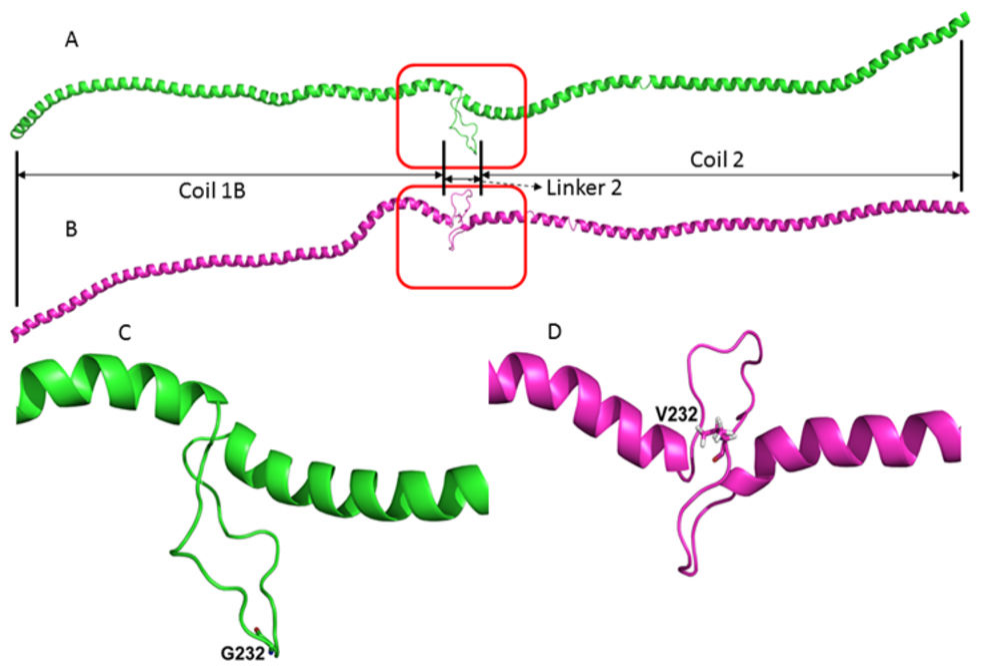
Structural models of LMNA. (**A**) The model of wild type LMNA (residues 81−383) was built using the HHblits-generated parameters from Modeller. (**B**) The G232V LMNA mutant model. (**C**) Higher magnification of (A). (**D**) Higher magnification of (B). G232 and V232 are shown as sticks. Modeling visualization was performed using PyMol (http://www.pymol.org).

Intermediate filament structures are characterized as a central alpha-helical coiled-coil rod domain consisting of three alpha-helical segments (1A, 1B and 2) bridged by two linker regions [25]. Notably, the G232V mutation in *LMNA* is located in the 1B−2 linker region, also referred to as linker 2, and is hypothesized to play a crucial rule in stabilizing the assembly of intermediate filaments [4,25]. Therefore, we sought to build a structural model containing this site. To accomplish this, we utilized HHpred to search for the structures with the highest sequence identity across this region: tropomyosin from *Sus scrofa* (PDB ID: 1C1G) [19], human lamin coil 2B (PDB ID: 1X8Y) [20], human vimentin coil 2B fragment (PDB ID: 1GK4) [21] and human vimentin coil 2 fragment (PDB ID: 3TRT) [22]. Structural models of LMNA were built using Modeller [24] with the HHblits-generated parameters. Glycine 232 is located at the tip of a loop in WT *LMNA* (Figure 3). Interestingly, a glycine-to-valine substitution at this site led to a dramatic conformational change, possibly because of the large valine side chain. Subsequently, this mutant form may assemble more loosely as a dimer or assemble into a dimer of a different configuration, which hinders the subsequent formation of the coiled-coil rod domain.

### Expression of the p.Gly232Val Mutation in the HL-1 Cell Line

**Figure 4:**
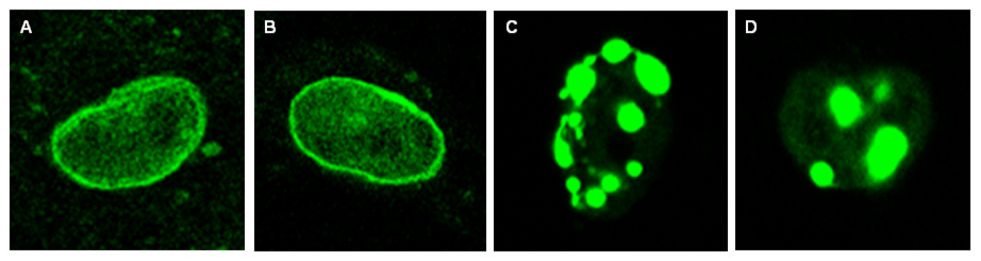
Expression of lamin A/C in cultured atrial cardiomyocytes. HL-1 atrial cardiomyocytes transfected with wild-type lamin A (**A**) and lamin C (**B**) in fluorescent expression vectors, showing the cell nucleus with homogenous nuclear veil. HL-1 atrial cardiomyocytes transfected with p.G232V mutant lamin A (**C**) and lamin C (**D**), showing the cell nucleus with aggregates.

Lamin A and lamin C constructs were transfected separately into HL-1 cells. As shown on Figure 4, cells transfected with WT constructs (Figure 4A for lamin A; Figure 4B for lamin C) displayed fluorescence homogeneously distributed at the inner nuclear lamina. After transfection of the p.Gly232Val mutated lamin A and lamin C constructs (Figure 4C for lamin A; Figure 4D for lamin C), we observed characteristic aggregates indicating an uneven distribution of lamin protein within the nucleus. 

## Discussion

We used whole-exome sequencing to evaluate a family with autosomal dominant CCD and identified a heterozygous missense mutation (NM_005572, c.G695T, G232V) in the *LMNA* gene as the most likely candidate mutation. The ANNOVAR program predicted that this mutation would be damaging, and the HHpred software predicted that it would affect the conformation of the LMNA coiled-coil rod domain. We further verified its pathogenicity by expressing the mutation in a cellular model. Subsequently, we confirmed that the mutation showed a complete cosegregation with the disease phenotype in the family; it was carried by all three affected individuals and was absent in the three healthy individuals. We further confirmed the absence of the mutation in 137 additional healthy controls and 115 patients with atrial fibrillation. 

More than 450 different mutations have been identified in the *LMNA* gene that can cause a variety of diseases [26], called laminopathies, and have been categorized into 4 major types of disorders: striated and cardiac muscle diseases, lipodystrophy syndromes, peripheral neuropathy and premature aging. Since Bonne et al. identified the first *LMNA* gene mutation in patients affected with autosomal dominant Emery-Dreifuss muscular dystrophy (EDMD), dilated cardiomyopathy (DCM) and conduction defects in 1999 [27], mutations in *LMNA* have subsequently been identified in individuals with isolated DCM, usually with a conduction defect [28]. Atrial or ventricular arrhythmias are often present and even precede the onset of DCM [28-33]. The heterogeneity of *LMNA* mutations was further established by the lack of clear genotype-phenotype correlations [26,34]. Interestingly, the major phenotype of our study family was isolated CCD characterized by extensive atrial disease, tachy-brady arrhythmias and an atrioventricular conduction block. Similar to a previous observation that isolated cardiac involvement was predominantly found in patients with *LMNA* mutations located in the *a*-helical rod domain [28], none of the affected family members presented with muscular dystrophy or other organ system involvement. However, none of them had evidence of cardiomyopathy and LV dysfunction. There was also no history of sudden cardiac death in the family members. In contrast, there was a report of a patient with EDMD [35] whose mutation was identical to the one found in the study family except that the mutation in the patient was G to A instead of T and an amino acid change from glycine to glutamate instead of valine. However, the ECG and echocardiography of this patient at 11 years of age was normal in that report. More recently, another study reported a patient with EDMD whose mutation (c.694GC) also affected the codon 232 and changed glycine to arginine. Interestingly, this patient had DCM, right bundle branch block and ventricular arrhythmia at 18 years of age in that report [36]. Thus, the clinical manifestations of *LMNA* mutations are complex and most likely reflect the interaction of background genetic modifiers and environmental factors for the development of the phenotype.

The pathogenic mechanism underlying the association between the *LMNA* mutations and laminopathies remains unclear. Our structural model prediction suggested a dominant negative effect resulting from the G232V mutation. The structural modeling of the *LMNA* mutation showed a conformational change in the linker 2 region, which may destabilize the rod of the natural homodimer. The linker 2 region of *LMNA* is homologous to the linker L12 regions in other intermediate filaments proteins [25]. One hypothesis [37] is that the conformational change alters the quaternary structure of intermediate filament proteins. The heterozygous *LMNA* mutation genotypes also suggest that the conformational change is caused by the formation of a heterodimer between the normal LMNA protein and the mutated protein. The pathogenicity of the G232V mutation predicted by the structure model was further supported by the fact that the transient transfection of the mutated cDNA in HL-1 atrial mocyteses leads to abnormal lamin aggregates in the nucleus and to the loss of internal nuclear lamin organization, which are in agreement with the findings of previous reports [38]. Previous autopsy studies revealed marked fibrosis and fatty metamorphosis of the sinoatrial and atrioventricular nodes as well as the atrioventricular bundle in patients with mutations in the *LMNA* gene and conduction defect [28,39]. Thus, more studies are needed to determine whether the G232V mutation could further cause lamina instability *in vivo* as well as subsequent cell apoptosis in the cardiac conduction system or the loss of lamin-dependent gene regulatory function that was previously reported in some *LMNA* missense mutations [40]. 

We did not attempt to perform linkage analysis on our study family because of the small pedigree size. We conducted direct sequencing analysis on the *SCN5A* gene in the affected father prior to this study because the sick sinus syndrome 1 gene is the sodium channel *SCN5A* gene, and we did not find a causal mutation in the *SCN5A* gene. With the development of whole-exome sequencing, we could obviate the extensive laboratory work and expenses incurred by subsequent targeted sequencing of many previously identified candidate genes. However, the main challenge of using exome sequencing to find novel disease genes for our study was distinguishing between disease-related alleles and non-pathogenic polymorphisms and sequencing errors. For Mendelian disorders, the use of pedigree information can substantially narrow the search. Interestingly, subsequent discrete filtering indicated the autosomal dominant inheritance of the trait. Although the discrete filtering method we adopted has been well-established and used successfully to identify disease-causing mutations, these filtering strategies do not remove false positive variants that cannot be logically eliminated by filtering alone [10]. This limitation could be overcome if linkage mapping data were available. Furthermore, the Holter recording and echocardiography studies on the young grandson, 31 years of age, whose phenotype was still not established, were normal. Although 92% of carriers with 30+ years of age had conduction disease, including atrioventricular conduction disturbances, atrial arrhythmias and ventricular arrhythmias in a meta-analysis of 299 patients with *LMNA* mutations [41], the possibility of a false-positive call for *LMNA* G232V still cannot be completely ruled out. Moreover, genetic heterogeneity has been observed in inherited CCD. False negative calls might occur in the regions of genes such as *SCN5A*, *HCN4*, *KCNJ2*, *NKX2-5*, *TBX5*, *Cx40*, *PRKAG2* and others that are known to be involved in CCD if the depth and quality of reads were insufficient to confidently discover a variant at that site. Finally, technical failure could occur when causative mutations occur in coding sequences outside the target region, such as in the intronic or regulatory regions or outside the gene.

Rare monogenic diseases are of substantial interest because the identification of their genetic bases provides important knowledge about disease mechanisms, biological pathways and potential therapeutic targets. Our studies support the previous observations that cardiac manifestation of laminopathies is heterogeneous, and the diagnosis of laminopathy should be suspected in a patient with an early onset of atrial fibrillation, progressive conduction disease with or without DCM and a family history of sudden cardiac death. The identification of *LMNA* as the candidate gene underlying CCD in the study family enables presymptomatic genetic diagnosis and early disease management. The affected siblings should be monitored closely for the development of DCM and lethal arrhythmias. Additionally, identifying a familial mutation can be valuable in reproductive planning and in the identification of family members who are not carriers of the mutation and will thus not require lifelong surveillance. Even if the young grandson turns out to be affected, we still can use the *ZFHX3* and *MMP2* family-specific mutations as markers associated with the trait for future monitoring.

Apart from the intrinsic limitations associated with a whole-exome sequencing study, our study has several other limitations. The main limitation is that our study was only analyzed in an *in vitro* functional manner and showed only an arguable relationship with the disease. However, there has been a lack of suitable research methods to study the molecular mechanisms linking cardiac laminopathy to electrophysiological abnormalities. We anticipate that future studies utilizing patient-specific induced pluripotent stem cells cardiomyocyts models will elucidate electrophysiological features of cardiac laminopathies. Second, the uncertain phenotype for the young grandson and the lack of genotypic information on the affected father throw into doubt the causal relationship between the *LMNA* mutation and the disease. Third, only mutations in genes previously linked to cardiovascular disease were considered. There is a possibility that the 13 genes with mutations (including 2 indels) not known as cardiovascular genes could potentially contribute to CCD in this family. Finally, replicating *LMNA* mutations in an independent family or a cohort of unrelated patients with CCD would improve the strength of the analysis.

In conclusion, we used whole-exome sequencing to evaluate a family with autosomal dominant CCD and proposed a missense mutation in the *LMNA* gene as the most likely candidate mutation. Our results implicated whole-exome sequencing as a feasible approach for the identification of candidate genes underlying inherited conduction diseases. 

## Supporting Information

Figure S1 **Deduced genotypes in the pedigrees according to the possible inheritance models.** “A” represents the dominant trait, and “a” represents the recessive trait. “X^A”^ represents the dominant trait on chromosome X, and “X^a^” represents the recessive trait on chromosome X. (**A**) Type I, autosomal dominant model. We considered the disease trait to be the dominant trait in the pedigree. (**B**) Type II, autosomal recessive model. We considered the disease trait to be the recessive trait in the pedigree. (**C**) Type IV: X-linked recessive model. We assumed that the disease trait was the recessive trait on the X chromosome in the pedigree.
(TIFF)

Table S1 **Primer sequences and restriction enzyme (RE) used in the analysis of novel variants.**
(DOCX)

Table S2 **Indel variants that pass the filtering criteria and match to the autosomal dominant pedigree.**
(DOCX)

Table S3 **SNVs that pass the filtering criterions and match to the autosomal dominant pedigree.**
(DOCX)
