# Supplementary material for: Whole-Exome Sequencing to Identify a Novel LMNA Gene Mutation Associated with Inherited Cardiac Conduction Disease
Source: PLoS One. 2013 Dec 12;8(12):e83322. doi: 10.1371/journal.pone.0083322 (PMC3861486; doi:10.1371/journal.pone.0083322)
Supplement: Table S1 — Primer sequences and restriction enzyme (RE) used in the analysis of novel variants. (DOCX) [file pone.0083322.s002.docx]

**Supplemental Table 1.** Primer sequences and restriction enzyme (RE) used in the analysis of novel variants

| Gene name | Primer sequence | PCR size (bp) | RE | Allele | Amino acid change |
| --- | --- | --- | --- | --- | --- |
| LMNA | (F)-5'-CCGACTGGTGGAGATTGACAAC-3', (R)-5'-GGAGGCACAGGCCTGAGTT-3' | 304 | HpyCH4IV | G>T | G232V |
| ZFHX3 | (F)-5'-CAATTGCACCGGCCCAGCCATT-3', (R)-5'-CGCAAGACTCGGAGCTGATCATCTGT-3' | 259 | AseI | G>A | V1169M |
| MMP2 | (F)-5'-GGAGAAGGCCAAGGTGAGAA-3', (R)-5'-GGTATCAGTGCAGCTGTTGTG-3' | 281 | Bsp1286I | G>A | E193K |
| ANGPTL5 | (F)-5'-GGAAATACCTCAAATGGGTAGCTA-3', (R)-5'-GGAAACTAATCACTAACAGTGACATT-3' | 342 | DdeI | C>T | P164L |
| MYH7B | (F)-5'-GGAAGAAGCAGGTGCAGAAGCTGTA-3' (R)-5'-CCTTGACACGGCGCTCATGCTT-3' | 209 | Hpy166II | G>A | A1866T |

RE: restriction enzyme
