# Supplementary material for: Whole-Exome Sequencing to Identify a Novel LMNA Gene Mutation Associated with Inherited Cardiac Conduction Disease
Source: PLoS One. 2013 Dec 12;8(12):e83322. doi: 10.1371/journal.pone.0083322 (PMC3861486; doi:10.1371/journal.pone.0083322)
Supplement: Table S2 — Indel variants that pass the filtering criteria and match to the autosomal dominant pedigree. (DOCX) [file pone.0083322.s003.docx]

**Supplemental Table 2**. Indel variants that pass the filtering criteria and match to the autosomal dominant pedigree

| Position | Gene Symbol | DNA Sequence variation | Protein sequence variation |
| --- | --- | --- | --- |
| Chr7:150935600 | CHPF2 | c.2152–2153insG | R718fs |
| Chr11:93844067~93844070 | HEPHL1 | c.3046–3047del AGAT | ∆I1016 |

CHPF2, chondroitin polymerizing factor 2; HEPHL1, hephaestin-like 1
