# Supplementary material for: Whole-Exome Sequencing to Identify a Novel LMNA Gene Mutation Associated with Inherited Cardiac Conduction Disease
Source: PLoS One. 2013 Dec 12;8(12):e83322. doi: 10.1371/journal.pone.0083322 (PMC3861486; doi:10.1371/journal.pone.0083322)
Supplement: Table S3 — SNVs that pass the filtering criterions and match to the autosomal dominant pedigree. (DOCX) [file pone.0083322.s004.docx]

**Supplemental Table 3.** SNV**s** that pass the filtering criterions and match to the autosomal dominant pedigree

| Func | Gene | ExonicFunc | AA Change | ESP5400 | 1000G 2012feb | PhyloP | SIFT | PolyPhen2 | LRT | MutationTaster | GERP++ | Chr | Position | Ref | Obs |
| --- | --- | --- | --- | --- | --- | --- | --- | --- | --- | --- | --- | --- | --- | --- | --- |
| exonic | BDH2 | nonsynonymous | NM_020139:c.G412A:p.V138I | 0.000093 | 0.01 | 0.998086 | 0 | 0 | 0.999999 | 1.77E-04 | 1.31 | chr4 | 104007643 | C | T |
| exonic | ARID2 | nonsynonymous | NM_152641:c.G4300T:p.A1434S | 0.000093 | 0.02 | 0.247155 | 1 | 0.813 | 0.993914 | 0.919633 | -0.289 | chr12 | 46246206 | G | T |
| exonic | MEP1B | nonsynonymous | NM_005925:c.C1268T:p.S423L | 0.0002 |  |  |  |  |  |  |  | chr18 | 29793211 | C | T |
| exonic | OR7E24 | nonsynonymous | NM_001079935:c.G419A:p.R140Q | 0.000279 | 0.0032 |  | 0 |  |  |  |  | chr19 | 9362138 | G | A |
| exonic | PHLDB1 | nonsynonymous | NM_001144759:c.G3472A:p.V1158I | 0.000279 | 0.01 | 0.999548 | 0.62 | 0.063 | 0.999986 | 0.705257 | 5.11 | chr11 | 118518751 | G | A |
| exonic | PMVK | nonsynonymous | NM_006556:c.G329A:p.R110Q | 0.000279 |  | 0.99722 | 1 | 0.999 | 1 | 0.999785 | 4.4 | chr1 | 154898943 | C | T |
| exonic | TREH | nonsynonymous | uc001ptz.1:c.T218C:p.M73T | 0.000288 | 0.01 |  | 0 |  |  |  |  | chr11 | 118532376 | A | G |
| exonic | DMBT1 | nonsynonymous | NM_004406:c.C3117A:p.D1039E | 0.000295 | 0.04 |  |  |  |  |  |  | chr10 | 124380676 | C | A |
| exonic | SLC39A7 | nonsynonymous | NM_006979:c.G697A:p.V233M | 0.000439 |  | 0.998262 | 0.94 | 0.993 | 1 | 0.999933 | 3.56 | chr6 | 33170102 | G | A |
| exonic | SRRT | nonsynonymous | NM_001128852:c.T697G:p.F233V | 0.000651 |  | 0.996163 | 0.75 | 0.734 | 1 | 0.999643 | 3.53 | chr7 | 100481800 | T | G |
| splicing | F2 |  |  | 0.001859 |  |  |  |  |  |  |  | chr11 | 46748178 | T | G |
| exonic | CACNA1D | stopgain | NM_001128839:c.C5529A:p.Y1843X | 0.007436 |  | 0.964193 | 0.913711 | 0.59086 | 0.212287 | 1 | 2.98 | chr3 | 53839025 | C | A |
| exonic | MED23 | nonsynonymous | NM_004830:c.T290G:p.V97G | 0.010134 |  | 0.998663 | 1 | 0.995 | 1 | 0.999632 | 5.21 | chr6 | 131944597 | A | C |
| exonic | CHD3 | nonsynonymous | NM_001005271:c.T4499G:p.V1500G | 0.015616 |  | 0.997667 | 1 | 0.785076 | 0.979656 | 0.999938 | 4.62 | chr17 | 7809271 | T | G |
| exonic | PGM2 | stopgain | NM_018290:c.C1422A:p.Y474X | 0.03032 |  | 0.858756 | 0.892586 | 0.712326 | 0.999978 | 1 | 2.99 | chr4 | 37851814 | C | A |
| exonic | NFS1 | nonsynonymous | NM_001198989:c.A293G:p.E98G | 0.032255 |  | 0.997756 | 1 | 0.96 | 1 | 0.999993 | 5.2 | chr20 | 34285637 | T | C |
| splicing | SPEG |  |  | 0.082026 |  |  |  |  |  |  |  | chr2 | 220342509 | T | G |
| exonic | CCDC155 | nonsynonymous | NM_144688:c.T161G:p.V54G | 0.118497 |  |  |  |  |  |  |  | chr19 | 49898375 | T | G |
| exonic | CYB561 | nonsynonymous | NM_001017916:c.T413G:p.V138G | 0.250186 |  | 0.998663 | 0.99 | 0.983 | 0.995623 | 0.993428 | 4.21 | chr17 | 61512597 | A | C |
| exonic | TSN | nonsynonymous | NM_004622:c.C662T:p.T221M |  | 0.0005 | 0.937631 | 0.9 | 0.001 | 0.99656 | 0.152607 | 3.13 | chr2 | 122522918 | C | T |
| exonic | SRSF12 | nonsynonymous | NM_080743:c.G536A:p.R179Q |  | 0.0005 |  |  |  |  |  |  | chr6 | 89808547 | C | T |
| exonic | ERBB2 | nonsynonymous | NM_004448:c.G808T:p.A270S |  | 0.0005 | 0.810779 | 0.49 | 0.003 | 0.844392 | 0.323425 | -0.261 | chr17 | 37866641 | G | T |
| exonic | TM6SF2 | nonsynonymous | NM_001001524:c.G919A:p.G307S |  | 0.0005 | 0.9985 | 0.86 | 0.883 | 0.994723 | 0.15193 | 4.99 | chr19 | 19377304 | C | T |
| exonic | KIF6 | nonsynonymous | NM_145027:c.C2342T:p.S781L |  | 0.0009 | 0.998888 | 0.99 | 0.143 | 0.851936 | 0.103367 | 4.12 | chr6 | 39311571 | G | A |
| exonic | TECTA | nonsynonymous | NM_005422:c.C2967A:p.H989Q |  | 0.0009 | 0.059837 | 0.83 | 0.999 | 1 | 0.306344 | -2.14 | chr11 | 121008155 | C | A |
| exonic | ACSF3 | nonsynonymous | NM_001127214:c.G1213A:p.A405T |  | 0.0009 | 0.999135 | 0.88 | 0.318 | 0.986577 | 0.877519 | 4.61 | chr16 | 89187295 | G | A |
| exonic | FBXO47 | nonsynonymous | NM_001008777:c.T1152A:p.F384L |  | 0.0009 | 0.919653 | 0.29 | 0.642 | 1 | 0.900529 | 1.2 | chr17 | 37094917 | A | T |
| exonic | OR11G2 | nonsynonymous | NM_001005503:c.T251A:p.I84N |  | 0.0014 | 0.996056 | 1 | 0.981 | 0.964388 | 0.015565 | 4.58 | chr14 | 20665745 | T | A |
| exonic | SPPL2C | nonsynonymous | NM_175882:c.G992T:p.C331F |  | 0.0014 | 0.999617 | 0.96 | 1 | 0.999927 | 0.953018 | 4.71 | chr17 | 43923264 | G | T |
| exonic | ANKRD11 | nonsynonymous | NM_001256183:c.A5665G:p.K1889E |  | 0.0027 | 0.995355 | 0.86 | 0.931 | 0.999998 | 0.013713 | 4.39 | chr16 | 89347285 | T | C |
| exonic | HIST1H2AK | nonsynonymous | NM_003510:c.A40C:p.K14Q |  | 0.0046 | 0.995992 | 0.99 | 0.557532 | 0.996711 | 0.010686 | 3.45 | chr6 | 27806078 | T | G |
| exonic | FAT3 | nonsynonymous | NM_001008781:c.G1067A:p.C356Y |  | 0.01 |  |  |  |  |  |  | chr11 | 92086345 | G | A |
| exonic | FAM135A | nonsynonymous | NM_001162529:c.G92A:p.R31H |  | 0.02 |  |  |  |  |  |  | chr6 | 71162209 | G | A |
|  |  |  |  |  |  |  |  |  |  |  |  |  |  |  |  |
| exonic | SLC39A1 | nonsynonymous | NM_014437:c.C317G:p.T106R |  |  | 0.999646 | 0.97 | 0.843 | 1 | 0.999119 | 4.76 | chr1 | 153934697 | G | C |
| exonic | LMNA | nonsynonymous | NM_005572:c.G695T:p.G232V |  |  | 0.990047 | 1 | 0.967 | 0.999987 | 0.999618 | 3.88 | chr1 | 156104651 | G | T |
| splicing | SACM1L |  |  |  |  |  |  |  |  |  |  | chr3 | 45781138 | T | G |
| exonic | ADH4 | nonsynonymous | NM_000670:c.G884A:p.C295Y |  |  | 0.152746 | 1 | 1 | 0.993274 | 0.990438 | 2.98 | chr4 | 100048455 | C | T |
| exonic | MFSD8 | nonsynonymous | NM_152778:c.T1116A:p.N372K |  |  | 0.127929 | 0.39 | 0.302 | 0.971215 | 0.994185 | -2.04 | chr4 | 128842913 | A | T |
| exonic | DLK2 | nonsynonymous | NM_023932:c.G1004A:p.G335D |  |  | 0.998046 | 1 | 0.992 | 0.999744 | 0.319812 | 4.74 | chr6 | 43418425 | C | T |
| exonic | MDN1 | nonsynonymous | NM_014611:c.G1303A:p.A435T |  |  | 0.983174 | 0.85 | 0.051 | 0.999992 | 0.07298 | 4.29 | chr6 | 90497604 | C | T |
| exonic | ODZ4 | nonsynonymous | NM_001098816:c.G7330A:p.V2444I |  |  |  |  |  |  |  |  | chr11 | 78380060 | C | T |
| exonic | ANGPTL5 | nonsynonymous | NM_178127:c.C491T:p.P164L |  |  | 0.999101 | 0.69 | 1 | 1 | 0.999707 | 4.59 | chr11 | 101773401 | G | A |
| exonic | L2HGDH | nonsynonymous | NM_024884:c.A1174G:p.I392V |  |  | 0.998448 | 0.57 | 0.206 | 0.999864 | 0.917279 | 5.25 | chr14 | 50732098 | T | C |
| exonic | ZNF646 | nonsynonymous | NM_014699:c.C2530T:p.P844S |  |  | 0.998086 | 0.29 | 0.997 | 1 | 0.672125 | 4.75 | chr16 | 31090175 | C | T |
| exonic | LONP2 | nonsynonymous | NM_031490:c.G2276A:p.R759Q |  |  | 0.999825 | 0.68 | 0.982 | 1 | 0.999344 | 5.82 | chr16 | 48382140 | G | A |
| exonic | MMP2 | nonsynonymous | NM_001127891:c.G577A:p.E193K |  |  | 0.998773 | 0.86 | 0.581 | 1 | 0.999997 | 4.35 | chr16 | 55519584 | G | A |
| exonic | ZFHX3 | nonsynonymous | NM_001164766:c.G3505A:p.V1169M |  |  | 0.998623 | 0.835347 | 0.417 | 0.999801 | 0.968961 | 5.05 | chr16 | 72830334 | C | T |
| exonic | MVD | nonsynonymous | NM_002461:c.G629A:p.G210D |  |  | 0.997123 | 0.99 | 0.077 | 0.999993 | 0.959602 | 4.37 | chr16 | 88722113 | C | T |
| exonic | KCNH6 | nonsynonymous | NM_030779:c.T1151G:p.V384G |  |  | 0.995344 | 0.86 | 0.989 | 1 | 0.999923 | 3.89 | chr17 | 61613079 | T | G |
| exonic | USF2 | nonsynonymous | NM_207291:c.C332A:p.T111N |  |  | 0.995125 | 0.94 | 0.011 | 0.996188 | 0.14421 | 3.36 | chr19 | 35761453 | C | A |
| exonic | MYH7B | nonsynonymous | NM_020884:c.G5596A:p.A1866T |  |  | 0.999257 | 0.806558 | 0.498668 | 0.980976 | 0.212551 | 4.2 | chr20 | 33588956 | G | A |
| exonic | ARHGAP40 | nonsynonymous | NM_001164431:c.G1507A:p.A503T |  |  |  |  |  |  |  |  | chr20 | 37272494 | G | A |

dbNSFP functional prediction scores integrated and normalized scores for PhyloP [prediction of a conserved (>0.95) or non-conserved (<0.95) site], SIFT [prediction of a change being damaging (>0.95) or tolerated (<0.95)], Polyphen2 [prediction of a change as damaging (>0.85), possibly damaging (0.85-0.15) or benign (<0.15)], LRT [likelihood ratio test for codon constraint ranging from 0–1; closer to 1 is more likely to be damaging], MutationTaster [prediction of a disease-causing variant, 1-p-value],) and GERP++ conservation scores [>0 indicates generally conserved].
